# A novel learning algorithm to predict individual survival after liver transplantation for primary sclerosing cholangitis

**DOI:** 10.1371/journal.pone.0193523

**Published:** 2018-03-15

**Authors:** Axel Andres, Aldo Montano-Loza, Russell Greiner, Max Uhlich, Ping Jin, Bret Hoehn, David Bigam, James Andrew Mark Shapiro, Norman Mark Kneteman

**Affiliations:** 1 Transplantation Surgery, Dept of Surgery, University of Alberta Hospital, Edmonton, Alberta, Canada; 2 Visceral Surgery and Transplantation, Dept of Surgery, Geneva University Hospital, Geneva, Switzerland; 3 Alberta Transplant Institute, University of Alberta, Edmonton, Alberta, Canada; 4 Hepatology, Dept of Medicine, University of Alberta Hospital, Edmonton, Canada; 5 Dept of Computing Science, University of Alberta, Edmonton, Canada; 6 Alberta Innovates Centre for Machine Learning, Edmonton, Canada; University of Illinois at Chicago College of Medicine, UNITED STATES

## Abstract

Deciding who should receive a liver transplant (LT) depends on both urgency and utility. Most survival scores are validated through discriminative tests, which compare predicted outcomes between patients. Assessing post-transplant survival utility is not discriminate, but should be “calibrated” to be effective. There are currently no such calibrated models. We developed and validated a novel calibrated model to predict individual survival after LT for Primary Sclerosing Cholangitis (PSC). We applied a software tool, PSSP, to adult patients in the Scientific Registry of Transplant Recipients (n = 2769) who received a LT for PSC between 2002 and 2013; this produced a model for predicting individual survival distributions for novel patients. We also developed an appropriate evaluation measure, D-calibration, to validate this model. The learned PSSP model showed an excellent D-calibration (p = 1.0), and passed the single-time calibration test (Hosmer-Lemeshow p-value of over 0.05) at 0.25, 1, 5 and 10 years. In contrast, the model based on traditional Cox regression showed worse calibration on long-term survival and failed at 10 years (Hosmer-Lemeshow p value = 0.027). The calculator and visualizer are available at: http://pssp.srv.ualberta.ca/calculator/liver_transplant_2002. In conclusion we present a new tool that accurately estimates individual post liver transplantation survival.

## Introduction

**Fig 1:**
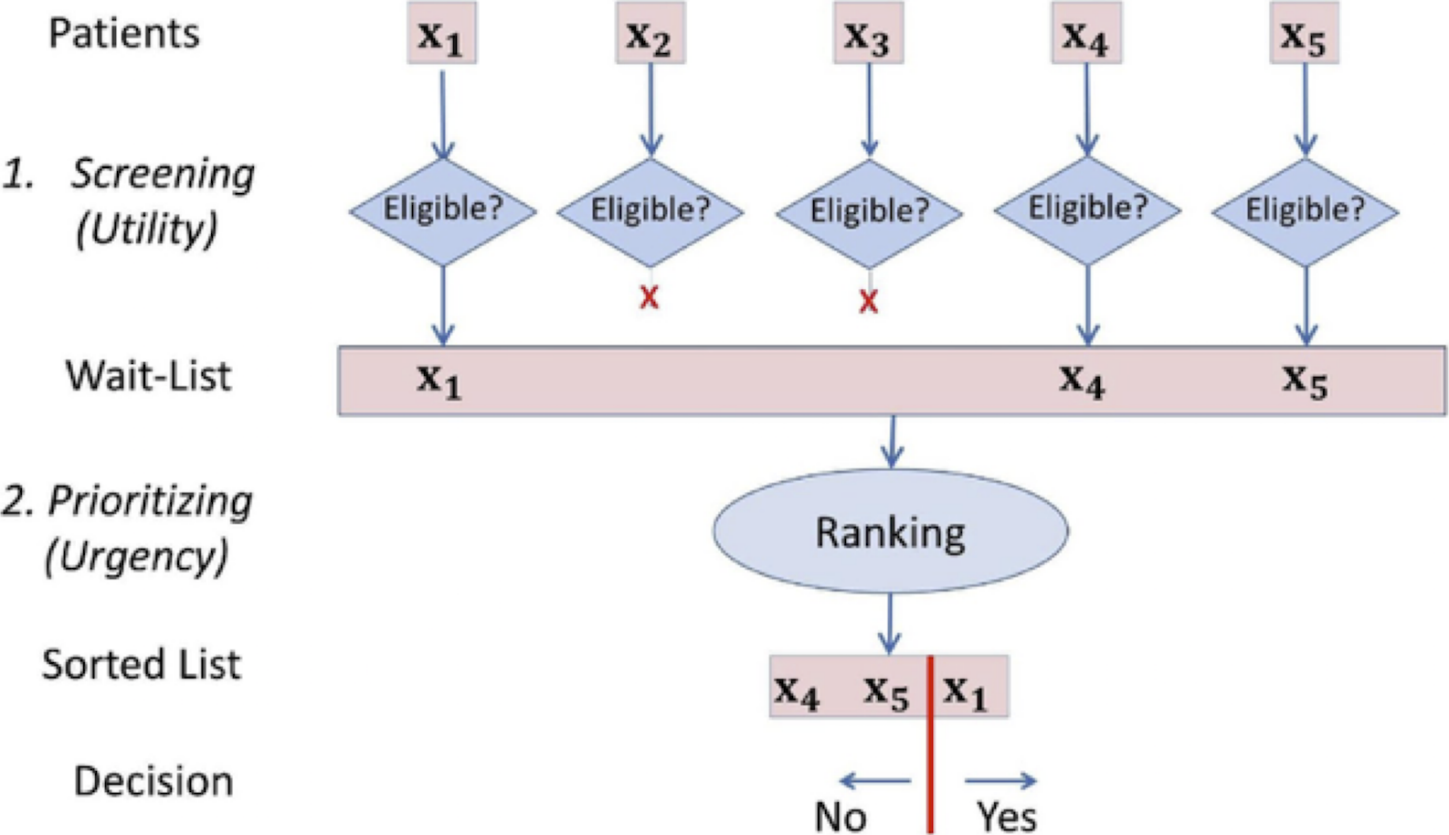
Two-step process. Two-step process for determining which patients should receive a Liver Transplant: Screening, then Prioritizing. This paper focuses on the initial “Screening” step.

Liver transplantation (LT) is the standard of care for selected patients with end-stage liver disease (ESLD) but is limited by organ shortage. To optimize the impact on survival of each available graft, transplant centres implicitly use the 2-step process shown in Fig 1:

1. An initial *Screening* process restricts the candidates based on anticipated survival, which corresponds to the utility of the graft. Note this decision is based *only on characteristics of this single patient*, and not in comparison to other patients.

2. A further *Prioritizing* step decides which patient on the wait-list should receive an available donor liver; this is decided competitively, based on urgency [1–3]–i.e., selecting the patient anticipated to die soonest without an LT.

To be as impartial as possible, scores are used to help clinicians in these processes. For Step 2 (Prioritizing), many transplant programs use the Model for End-Stage Liver Disease (MELD) score, which addresses this task, as it can predict which of 2 (or more) patients on the wait-list will die first; its effectiveness is reflected by its high concordance (c-)index (0.8) [4]. In contrast, no accurate model exists for Step 1 (Screening) of post-LT survival; many proposed models are considered of low accuracy because of low c-index [5–8].

While the Prioritization task is competitive (in that an available organ can only go to a single patient), the Screening task is based on the post-LT survival utility, which depends on the likelihood of survival for the individual patient, independent of the survival of other patients [9]. This means a Screening model is effective if its likelihood estimates are accurate; this effectiveness should be assessed by a calibration test, not a discriminative one, which means c-index is not relevant here (see Paragraph A in S1 File). The cut-offs defining appropriate survival likelihood are not established and vary between centers. Note that this utility should be based on more than a single time point (e.g. 1 year and 5 years), and ideally should take into account the whole predicted survival curve.

These realizations led to our Patient-Specific Survival Prediction (PSSP) system [10]: a tool that uses survival information from a database of earlier patients to learn a calibrated model. This trained model then uses a description of a new patient to produce an entire survival curve specific to this patient (Figure A in S1 File).

Here we apply PSSP to a cohort of LT patients with primary sclerosing cholangitis (PSC), a chronic cholestatic liver disease where inflammatory biliary strictures lead to cirrhosis and ESLD [11]. Now LT is the treatment of choice for such ESLD, with 5-year survival > 85% [12]. We focussed on this PSC prediction task as this high survival rate means it will be especially challenging–i.e., we anticipate that an approach that works here will also be effective for the other (less challenging) ESLDs.

We developed two PSSP models for predicting survival after a LT for PSC–the main text focuses on the model that uses information about the recipient alone, and the Supporting Information presents another model using both recipient and donor information—and created a public online calculator to allow users to easily apply these models to new patients. We also provide an appropriate way to validate the effectiveness of our model in estimating utility: “Distribution (D)-Calibration”.

## Patients and methods

This study used data from the Scientific Registry of Transplant Recipients (SRTR). The SRTR data system includes data on all donor, wait-listed candidates, and transplant recipients in the U.S., submitted by the members of the Organ Procurement and Transplantation Network (OPTN). The Health Resources and Services Administration (HRSA), U.S. Department of Health and Human Services provides oversight to the activities of the OPTN and SRTR contractors. The data reported here have been supplied by the Minneapolis Medical Research Foundation (MMRF) as the contractor for the SRTR. The interpretation and reporting of these data are the responsibility of the author(s) and in no way should be seen as an official policy of or interpretation by the SRTR or the U.S. Government. All data were fully anonymized before we accessed them. This study was approved by the Institutional Health Research Ethics Board, University of Alberta. The raw data utilized in this study are third party and can not be shared publicly because the rights to the data are retained by the U.S. Department of Health and Human Services/Health Resources and Services Administration and the SRTR Contractor. Data are available from the Scientific Registry of Transplant Recipients (SRTR); data requests can be sent to srtr@srtr.org.

Patient involvement: All the patients involved in this study were included on the waitlist and transplanted in centres members of the OPTN. As such, patients’ data were recorded according to the rules of the OPTN. The Minneapolis Medical Research Foundation approved data release by the SRTR. Centres that provided the data to the SRTR were not involved in the design of the study. This study focused on survival analysis after transplantation, independently of patients’ preference or priorities. None of the persons involved in this study were involved in patient recruitment and care.

We focused on overall survival after a first LT. Death was considered an event; otherwise patients were censored at time of last follow-up.

**Fig 2:**
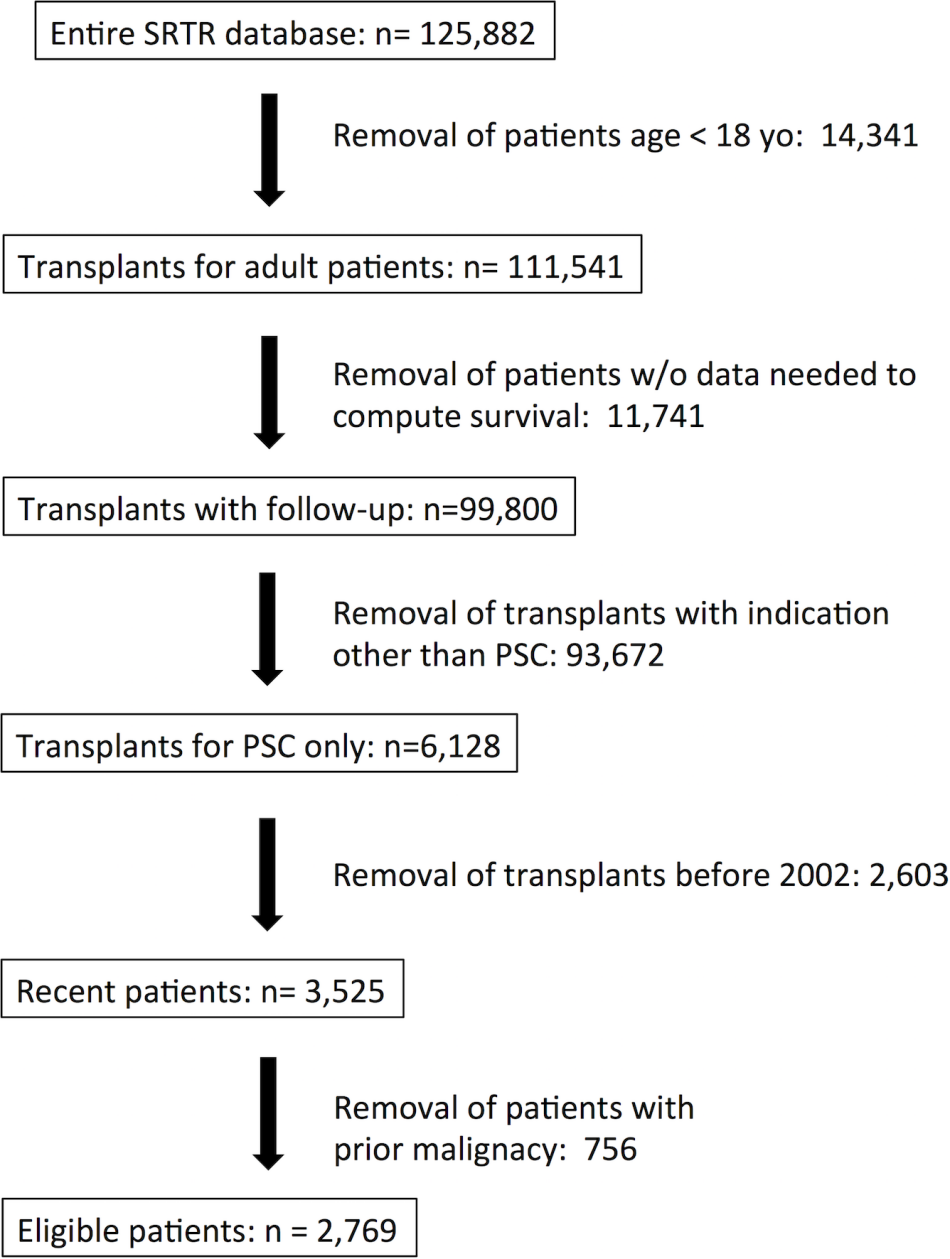
Patients’ selection process. Flow-chart describing the number of excluded (and remaining) patients at each stage of the selection process.

We included all adult (≥18 years old) PSC patients who underwent a first LT between January 2002, and November 2013. Exclusion criteria were overlap diagnoses at time of LT (primary biliary cirrhosis, autoimmune hepatitis, and others–based on explant histopathology), malignancies prior to LT, and patients who received more than two LTs. The overall selection process appears in Fig 2.

We considered all available clinically relevant variables, including many that have shown an impact on post-LT survival and/or were part of pre-existing scores such as MELD, Child-Pugh, and Donor Risk Index [13]. We excluded all variables whose values were missing in >40% of cases. Hence, our learning algorithm considered the following recipient variables at time of LT: gender, age, ABO blood group, INR, bilirubin, creatinine, MELD score, albumin, height, weight, BMI, history of Crohn's disease, history of ulcerative colitis, patient on ventilator, presence of ascites and/or hepatic encephalopathy, history of diabetes, and location of the patient before LT (home, hospital ward, ICU). We excluded patients with missing or problematic data mandatory to calculate survivals (e.g. aberrant dates or unclear alive/death status); see Fig 2.

We capped INR at 5, and MELD at 40, and set creatinine to 4.0 mg/dL if the candidate was dialyzed within a week. We included both natural and logarithmic forms of the following laboratory variables: INR, bilirubin and creatinine. We used mean imputation to fill in any missing values.

### The PSSP learning algorithm

PSSP uses data from a cohort of patients with a particular condition to produce a ‘survival model’, which can then be used to produce an individual “survival curve” for a new patient x_i_ (with this condition), based on the values of his/her variables. This curve resembles a Kaplan-Meier (KM) curve, as it provides, for each time *t* after the LT, the probability that this specific patient *x*_*i*_ will survive at least this long: P(death > *t* | *x*_*i*_); see Figure A in S1 File. In essence, PSSP first learns several logistic regression functions, one for each of a small number of time points {t_1_,.., t_k_}, each corresponding to the probability that a patient with these variable values will survive at least t_j_ days. It then combines these predictors, to ensure that the overall survival probability P(death > *t* | *x*_*i*_*)* is monotonically decreasing with time *t* [10]. See Paragraph B in S1 File.

### Variable selection

Including a high number of variables can lead to overfit models that do not generalize well to novel patients [14]. We therefore used the standard Cox variable selection process as a pre-processing step. This first used a univariate proportional hazard ratio test to identify the variables that were associated with survival time, retaining only the variables with p≤0.1. It then used an automated multivariate Cox regression to the estimate covariate-adjusted HR (Hazard Ratio) of the remaining variables, sequentially removing any that did not appear significant. We then built the PSSP model using only the remaining variables.

We also ran only the multivariate Cox filter (without the initial univariate Cox filter), and found this produced exactly the same set of variables.

### Evaluation

Survival models can be used for discriminative and/or calibration tasks. C-statistics [15] can evaluate any discriminative model that produces a single numeric “risk” score for each patient, by considering each pair of “comparable” patients, and asking whether the model’s values for these patients, matches what happened (see Paragraph D in S1 File).

This “c-statistics evaluation” is appropriate when the purpose of the survival model is discriminative, such as the Prioritizing step (Fig 1). The “Screening” step, however, makes independent decisions about each patient; this requires accurately knowing the probability that a specific patient *x*_*i*_ will survive at least a fixed time t (say *t* = 5 years, or *t* = 0.25 years)–i.e., computing an estimate $\hat{P}(t\;|x_{i})$.

There are many survival models that produce such estimates $\hat{P}(t_{0}\;|x_{i})\;$over different patients *x*_*i*_ for a *single time point t*_*0*_.These models are often evaluated using a Hosmer-Lemeshow test, to determine if they are “single time-point calibrated”–which we call “1-Calibrated” (see Paragraph E in S1 File) [16]. Here p <0.05 suggests the model is not 1-calibrated, while values close to 1 suggest good 1-calibration. We will use this evaluation below, for 0.25, 1, 5 and 10 years post-LT.

Note this “1-calibration” test is useful if the decision is based on *only a single time point*. However, the wait-list decision may well depend on several time points—both 0.25 and 5 year survival probabilities, and possibly other times as well.

**Fig 3:**
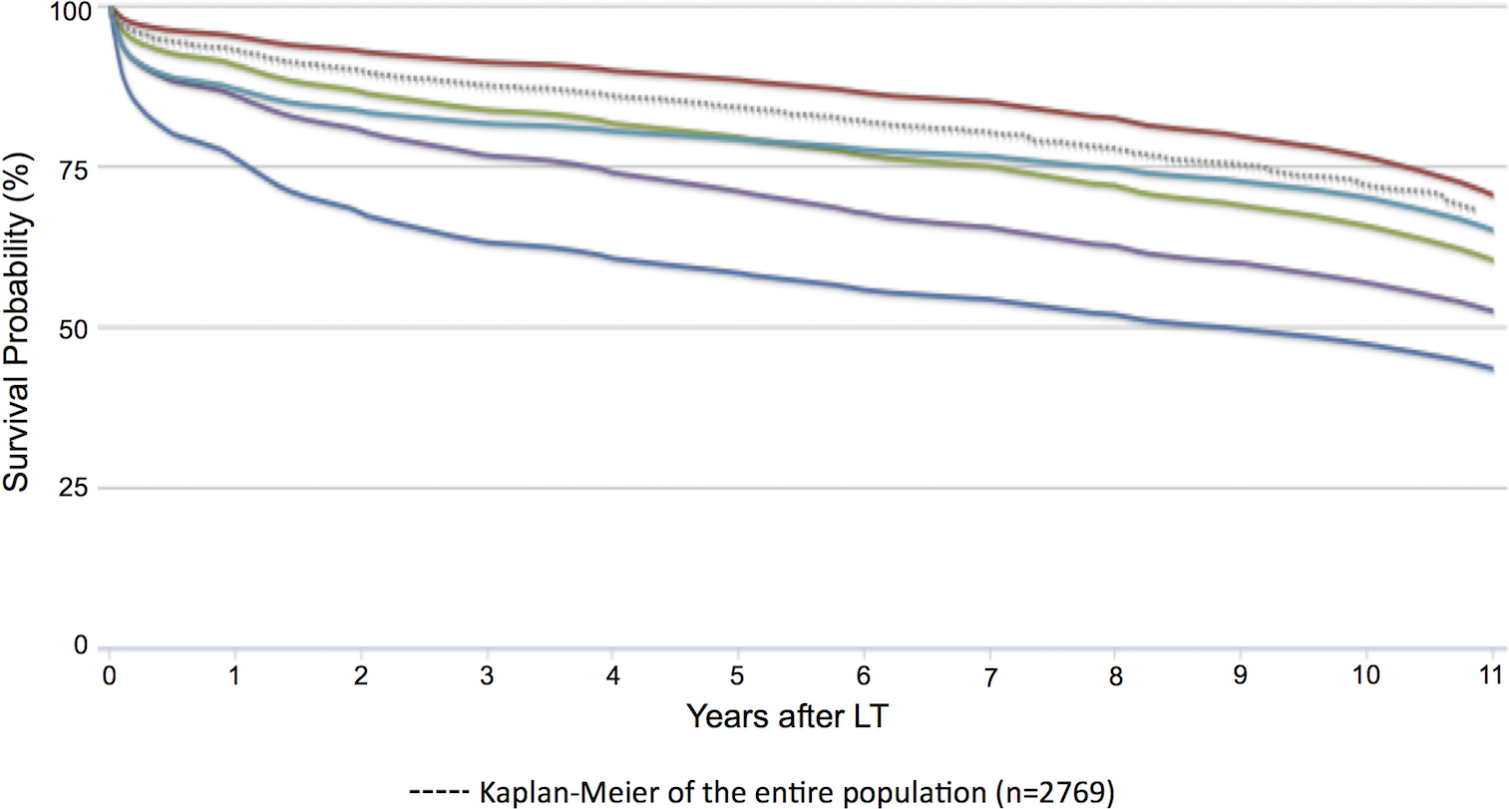
PSSP curves. Example of 5 representative curves, produced by PSSP after a first liver transplantation (solid curves). Each corresponds to a specific patient transplanted for PSC. The dashed curve corresponds to the Kaplan-Meier survival curve of the entire population of PSC patients (n = 2769 patients).

This motivates us to estimate the patient’s entire survival distribution–showing the probability that patient *x*_*i*_ will live at least time *t*, $\;\hat{P}(t\;|{\;x}_{i})$, over all time-points *t*. We represent this as a survival curve (Fig 3). This provides *x*_*i*_’s survival probability $\hat{P}(t\;|{\;x}_{i})\;$for each and every post-LT time *t*. To test the effectiveness of these curves, we could run the 1-calibration test (Hosmer-Lemeshow test) for each of several times, but it is not clear how many time-points to consider, nor how to combine these results to produce a single test. This suggests we should use an alternative test here–leading to “distributional-calibration” (“D-calibrated”).

To explain this test, first consider KM curves, which apply to an entire population. As an obvious test for calibration: over a set of patients, see how many died before the median. If this curve is D-calibrated, we expect 50% of these patients will die before their common predicted median survival time, and similarly we expect 75% of the patients to be alive at the 25th percentile, and so forth (see Paragraph F in S1 File, and Figure C in S1 File).

This is not possible for our *patient-specific* curves, as only a single patient is associated with each curve. However, we have thousands of PSC patients with individual curves and associated median survivals; we can therefore ask how many patients died before their *respective predicted median*? In a D-calibrated model, ½ of the patients should die before their median time. That is, if we let *d*_*i*_ represent the time when the i-th patient died, we are considering the set of $\left\{\hat{P}(d_{i}\;|{\;x}_{i})\;\right\}$ values over all the patients. Each number is in [0,^1^]; here we expect ½ of these values to be less than 0.5 –i.e., ½ will be in the interval [0, 0.5]. We are actually using 10 bins here, and so expect 10% of these $\left\{\hat{P}(d_{i}\;|{\;x}_{i})\;\right\}$ values to be in the [0.9, 1.0] interval, and another 10% in [0.8, 0.9], and so forth, down to the 10-th 10%, in [0.0, 0.1]. We then run a *χ*^*2*^-test over this data, to determine whether the model appears D-calibrated. Here, p <0.05 suggest the learned model is not D-calibrated, while a value of 1.0 indicates perfect D-calibration. Paragraphs F to H provide more details, and discusse how this test deals with censored instances).

As Cox models [17] are one of the most common survival models, we compare its accuracy to PSSP’s. Here, we use the Kalbfleisch / Prentice [18] way to estimate the base hazard function; we can combine this with the patient’s Cox risk score to estimate that patient’s individual survival function. We then compare this model (called “Cox-KP”) to the PSSP model, based on the same set of 1-calibration and D-calibration tests. All tests were run in 5-fold cross-validation. (To produce accurate estimates, we ran the variable-selection in-fold.)

To determine if the results were biased by systematic errors of coded entries by the centers, we also partitioned the 121 centres in the SRTR into 5 disjoint sets, and trained on ⅘ of these sets, and tested on the remaining set. We did this 5 times—so here we consider 5-fold cross-validation with regard to the centers, rather than the patients.

### Related prediction models

We created two survival models: 1. The main text presents the model based on only recipient’s variables. 2. The S1 File presents a model based on both donor’s and recipient’s variables.

### Calculator

As the model generated by PSSP cannot be expressed as a simple equation or nomogram (see Paragraph C in S1 File), we produced an online, publicly-available calculator that can produce either of these survival curves for a patient—either with or without donor information.

## Results

All results below relate just to the model without donor information, using the variables available at the time of the wait-listing. Paragraph J in S1 File provides information about the model with donor information.

### General

Inclusion criteria were met by 2769 patients; see Table 1. The mean follow-up time was 1658.18 days (SD 1237.60). The overall KM survival curve is presented in Fig 3 as the dashed curve, with 0.25, 1, 3, 5 and 10-year survival probabilities: 95.6%, 93%, 87.6%, 84.1% and 72%.

**Table 1:** Demographics of the included patients.

		At transplant	Multivariate analysis			
		[number of missing cases]		Confidence interval		
			HR	Lower	Upper	P
Number of patients		2769				
Follow-up time in days (mean, SD)		1658.18 (1237.60)				
Recipient Age in years (mean, SD)		47.41 (13.54) [0]	1.27	1.15	1.40	<0.001
Recipient Gender (M:F)		1923:846 [0]				
Recipient medical condition	• Hospitalized in Intensive Care Unit	186				
	•Hospitalized not in Intensive Care Unit	401				
	• Not hospitalized	2157 [25]	0.79	0.72	0.85	<0.0001
Recipient on ventilation support (No:Yes)		2709:60 [0]				
Recipient Diabetes (No:Yes)		2440:297 [32]	1.16	1.07	1.26	0.003
Presence of ascites before Tx (No:Yes)		871:1890 [8]				
Presence of encephalopathy (No:Yes)		1402:1359 [8]				
Last MELD score before Tx (mean, SD)		20.71 (8.58) [4]				
Last INR before TX (mean, SD)		1.64 (0.70) [4]				
Last Bilirubin before Tx (mean, SD)		11.70 (11.62) [3]				
Last Creatinine before Tx (mean, SD)		1.26 (1.08) [3]				
Last Albumin before Tx (mean, SD)		2.97 (0.74) [3]	0.88	0.81	0.97	0.03
Recipient weight in Kg (mean, SD)		76.17 (16.03) [79]				
Recipient height in cm (mean, SD)		173.89 (10.00) [102]				
Recipient Body Mass Index (mean, SD)		25.16 (4.71) [122]				
Recipient Inflammatory Bowel Disease (No:Yes)		1025:1744 [0]				
Recipient Crohn (No:Yes)		2340:429 [0]				
Recipient Ulcerative Colitis (No:Yes)		1425:1344 [0]				
Donor age in years (mean, SD)		40.35 (16.80)				
Donor Gender (M:F)		1599:1170 [0]				
Donor weight in Kg (mean, SD)		78.59 (19.07) [24]				
Donor height in cm (mean, SD)		171.64 (10.80) [41]				
Recipient ABO Group	A	1084 [0]				
	B	339 [0]				
	O	1237 [0]				
	AB	109 [0]				
Donor ABO Group	A	1041 [0]				
	B	311 [0]				
	O	1346 [0]				
	AB	71 [0]				
ABO compatibility	Compatible	169 [0]				
	Identical	2583 [0]				
	Incompatible	17 [0]				
Donor Type	Cadaveric	2387 [0]				
	Living	382 [0]				
Center experience in living donors (≤15 : >15)		243:139 [0]				
Donor Type	Brain dead	2292 [0]				
	Cardiac dead	95 [382]				
Donor cause of death	Anoxia	405 [0]				
	Cerebrovascular/stroke	961 [0]				
	Head trauma	963 [0]				
	Other	58 [0]				

The table includes the "Multivariable analysis" values (last 4 columns) only for the 4 variables considered significant by the subsequent multivariable analysis.

### PSSP curves

PSSP computed individual predicted survival curves for all 2769 patients (for each patient, this is based on the model trained on the other folds). Fig 3 presents 5 representative curves, along with the KM of the entire population.

### Variables selection

The variable selection algorithm had access to all the variables present at LT. Table 1 summarizes all variables that were considered, and notes which remained after the univariate and multivariate filters. We also show how many times each variable was missing. Note the hazard ratios for each selected variable is only indicative, as PSSP can allow one variable to have different impact at different times.

### Validation

#### D-Calibration

**Fig 4:**
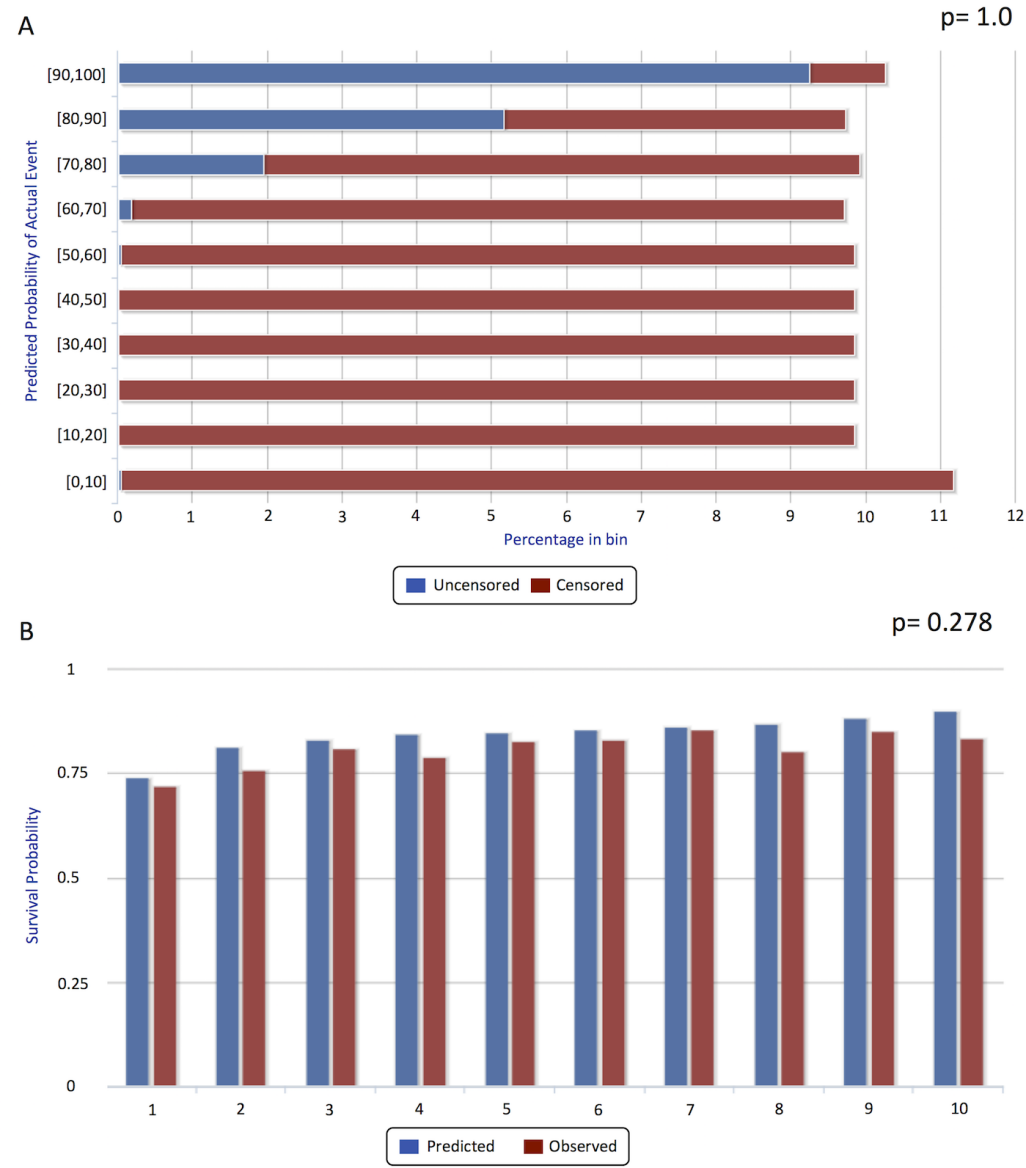
Distribution-calibration and single-point calibration (1-calibration). Panel A shows the observed distribution of events (death) histogram for each predicted decile of the survival distribution. The “p-value” here (1.0) is the result of the χ^2^test. Panel B shows the 5-years post post-transplant goodness-to-fit calibration (a.k.a. 1-calibration) histogram. Blue bars correspond to predicted and red bars to observed events, for each deciles of risk category according to the model. The p-value is 0.278, suggesting good calibration (Hosmer–Lemeshow).

The visual D-calibration of the model is represented as sideways histograms in Fig 4A. The χ^2^ test’s p-value of the D-calibration was 1.0 (S.D. 0), indicating excellent distributional calibration. The D-calibration p-value, assessed by cross validation, using partitioned centers was 0.999, confirming the absence of “center effect”.

### Single-timepoint validation (“1-calibration”)

Fig 4B shows the survival deciles for 5 years post-LT for the PSSP model, comparing the predicted versus the observed survival probabilities for each decile group. The Hosmer-Lemeshow p-values at 3 months, 1, 5 and 10 years for PSSP were 0.29, 0.13, 0.28 and 0.41, respectively; the corresponding 1-calibration p-values of the Cox-KP model were: 0.38, 0.32, 0.19 and 0.027; see Table A in S1 File. While both PSSP and Cox-KP model were acceptable at 3 months, 1 and 5 years, Cox-KP was not 1-calibrated at 10 years while PSSP still showed a good 1-calibration.

### Calculator

**Fig 5:**
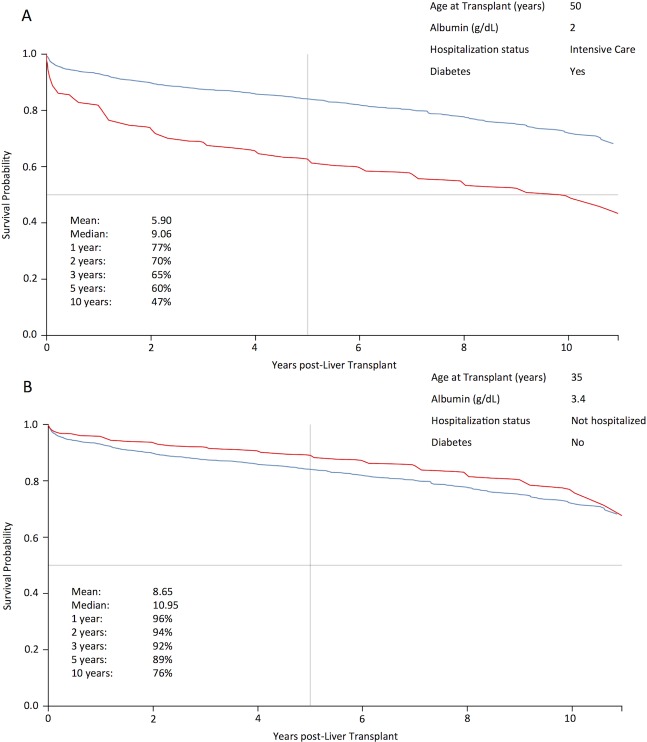
Two examples of predicted individual survival curve (in red) generated by the calculator. The blue curve corresponds to the Kaplan-Meier survival curve for the entire population used to learn the model (2769 patients). Of note, a vertical bar representing 5-years post-transplant survival shows a survival probability of 60% for the first patient (which should raise the question of the utility of such a transplant), and of 89% for the second patient, which is excellent in comparison to most indications for LT.

The model generated is available at: http://pssp.srv.ualberta.ca/calculator/liver_transplant_2002. Fig 5 provides examples of predicted survival curves for two different patients.

## Discussion

This paper describes three main results: 1. A novel tool for survival prediction, PSSP; 2. A novel (appropriate) method to evaluate individual survival curves, D-calibration; and 3. A demonstration that this PSSP tool works effectively on the task of predicting post-LT survival time for PSC patients.

Below we discuss the variables selected and compare our PSSP model to the more common risk models (such as Cox proportional hazard).

### Variables selected

We used the standard Cox filtering approach to select variables. This pre-processing step led to a model that used only four variables, and as expected, focused on variables already used in other LT analyses. High recipient age is associated with worse survival in PSC and is included in the Mayo, SOFT and BAR scores [7, 8, 19]. Other measures of severity, such as the need for hospitalization on a medical ward or intensive care unit (ICU) modify the risk of early post-transplant mortality, independent of MELD score [3, 20]. Low serum albumin level is associated with worse post-LT outcome [21] and reflects the nutritional status, which is an additional factor of post-LT morbidity [22]. Diabetes is an independent predictor of poor post-LT survival [23].

The MELD score, as well as its 3 component variables, were available to the learning algorithm, but none were found to be statistically significant. This result is not surprising: while MELD has proven effective in predicting urgency [24], it is poor in predicting post-LT survival [25, 26].

### PSSP vs “Risk score”

Many tools, including the simple Cox proportional hazard model, provide a risk score for each individual patient–a single value with the intent that patients with larger risks should die earlier than patients with smaller risks. These scores are useful for discriminative tasks, such as the Prioritization in Fig 1. They are not useful for our Screening task, as we need to know the actual P (*t* | *x*) value, for various times *t*, for a patient *x*–e.g., to decide whether P (5 years | x) > 0·.75. The risk scores, themselves, do not provide this P (t | x) information. (See Paragraph A in S1 File).

Additionally, most risk scores do not depend on time: If a model predicts that x_a_ is less likely than x_b_ to survive for 3 months, then it must also predict that x_a_ is less likely to survive for 5 years. PSSP, in contrast, can allow the hospitalization status to influence the chance of dying in the months immediately after LT, but be irrelevant at 5 years; it can also allow diabetes to have minimal peritransplant influence, but have a major impact on 5 year survival. This is essential in LT, where factors can influence the post-operative survival at different times. Note that Cox-KP inherits Cox’s “constant variable importance”.

While c-statistics (Table A in S1 File) reveals that both Cox and PSSP have mediocre discriminative capacity, recall that discrimination is not the goal for our screening test, but is instead calibrated survival prediction.

We anticipate this general PSSP approach will be applicable widely in the transplantation field and beyond–to any situation when we need to predict a patient’s future chance of survival. For example, LT for hepatocellular carcinoma should be performed only for those patients who have a high chance of survival. Many use the Milan Criteria [27] to identify patients whose survival is above 70% at 5 years post-LT. However, it only uses certain cancer factors; while they anticipate these factor will be dominant, they are not the only predictors of post-LT survival. It would be better to use PSSP here, to learn from these, and other patient variables, a model that accurately estimates survival probabilities, at this 5 year time, and also at other times.

This study focused on the LT Screening task–determining which PSC patients should be added to the wait-list, based on the utility of LT for each specific patient. We first note that this depends on the estimated survival probability, for each candidate patient, at several times. As this is a calibration task, not a discrimination task, the standard c-index measure is not appropriate for evaluation. This motivated us to design a measure that embodies “utility”, for evaluating such survival curves–“D-calibration”. We then introduce a new tool, PSSP, that can learn a general “survival model” from a survival dataset (here of PSC patients), which can then automatically produce survival curves for novel patients.

We ran this PSSP-learner on 2 tasks: with or without donor information. Our empirical results show that the individual survival distributions produced by these models are well calibrated, which means they can be used for this screening task of deciding whether a candidate should be added to the LT waitg list as they can help predict the survival of a possible recipient (or of a donor/recipient pair).

We also compared the calibration scores of our novel PSSP with the established Cox-KP system, and found that PSSP showed better calibration than Cox-KP at longer times–and in particular, that Cox-KP failed at 10 years post-LT, while PSSP was acceptable at all 4 times considered.

These results show that PSSP can accurately estimate the survival probability over time for an individual undergoing a complex intervention, based on a model learned on a survival database of prior patients with the intervention.

This technology can be applied to any medical situation where one needs to accurately estimate survival distributions for individual patients and would help us move toward evidence-based medicine on an individual level.

## Supporting information

S1 File Step-by-step explanation of PSSP and additional prediction model. A step-by-step explanation of the Patient Specific Survival Predictor, including management of censored patients, and prediction model including donor variables that are available at the time of the organ offer. Figure A in S1 File: Basic Machine Learning approach: Top-to-bottom: produce a PSSP model from a dataset of historical patients. Left-to-right (across bottom): producing a survival curve for a novel patient, using a description of that patient (that includes only the selected variables), based on the learned PSSP Model. Figure B in S1 File: Example of individual survival curves. Each of the 5 solid lines corresponds to the survival distribution, produced by PSSP, of a single patient using the model including donor variables. The dashed curve is the Kaplan-Meier plot over the entire population (n = 2769 patients). Figure C1 to C6 in S1 File: Step-by-step example to illustrate the concept of distribution-calibration. Figure D in S1 File: Sideways histogram, to visualize D-calibration of the model including donor variables. The “p-value” here (0.999) is the result of the *χ*^*2*^ test, on these values. Table A: Summary of the discrimination (Concordance) and calibration (1-calibration, D-calibration) tests for PSSP and Cox-Kalbfleisch-Prentice models, with and without donor information.
(DOCX)

## Abbreviations

SRTR: Scientific Registry of Transplant Recipients
PSSP: Patient-Specific Survival Prediction
PSC: Primary Sclerosing Cholangitis
LT: Liver Transplantation
ESLD: End-stage Liver Disease
MELD: Model for End-Stage Liver Disease
OPTN: Organ Procurement and Transplantation Network
HRSA: Health Resources and Services Administration
MMRF: Minneapolis Medical Research Foundation
ICU: Intesive Care Unit
KM: Kaplan-Meier
D-calibration: Distribution calibration
