# Supplementary material for: A novel learning algorithm to predict individual survival after liver transplantation for primary sclerosing cholangitis
Source: PLoS One. 2018 Mar 15;13(3):e0193523. doi: 10.1371/journal.pone.0193523 (PMC5854273; doi:10.1371/journal.pone.0193523)
Supplement: S1 File — A step-by-step explanation of the Patient Specific Survival Predictor, including management of censored patients, and prediction model including donor variables that are available at the time of the organ offer. Figure A in S1 File: Basic Machine Learning approach: Top-to-bottom: produce a PSSP model from a dataset of historical patients. Left-to-right (across bottom): producing a survival curve for a novel patient, using a description of that patient (that includes only the selected variables), based on the learned PSSP Model. Figure B in S1 File: Example of individual survival curves. Each of the 5 solid lines corresponds to the survival distribution, produced by PSSP, of a single patient using the model including donor variables. The dashed curve is the Kaplan-Meier plot over the entire population (n = 2769 patients). Figure C1 to C6 in S1 File: Step-by-step example to illustrate the concept of distribution-calibration. Figure D in S1 File: Sideways histogram, to visualize D-calibration of the model including donor variables. The “p-value” here (0.999) is the result of the χ2 test, on these values. Table A: Summary of the discrimination (Concordance) and calibration (1-calibration, D-calibration) tests for PSSP and Cox-Kalbfleisch-Prentice models, with and without donor information. (DOCX) [file pone.0193523.s001.docx]

**Supporting Information**

**Overview of Patient-specific survival prediction (PSSP)**

### A. Motivation

We noted earlier that the MELD score works well for Prioritizing candidates for liver transplantation (step 2 of Figure 1 in the main text). Trials that apply MELD to the Screening process of potential candidates for LT (step 1 of Figure 1 in the main text), however, have yielded low c-indices and so have been considered failures. This is problematic for two reasons.  First, the c-index is *not* the relevant evaluation here, as we want an absolute decision about this individual patient, not a relative (discriminative) comparison between pairs of patients.  Second, this 2^nd^ step requires the *survival probabilities* at different times, but there is no direct way to use the MELD score to estimate this information (as the score only refers to a severity scale).

That is, the Screening test relates to *calibration*, rather than *discrimination*. This has motivated many research projects to “cluster” patients into a small number of patient-groups (based on type of liver disease, co-morbidities or donor variables, etc.) then provide a Kaplan-Meier curve for each group, which can then provide the survival probability at a certain time (e.g. 5 years), which can be used to make a decision for the patient. However, this mandates the same decision (accept or reject) for every patient in each group (e.g., accept all patients with a specific End Stage Liver Disease but reject all with another), which ignores the wide heterogeneity within these sub-populations. As such, transplant candidate selection committees typically use empirical heuristics to inform their decision, attempting to evaluate the transplant benefit based on other relevant factors, including the patient’s age; disease process, severity and stage; comorbidities; prior surgery; etc.

This largely subjective process could be improved by a more objective system that uses all available evidence from thousands of previous patients to make a decision about *each individual patient*. This suggests using a learned survival curve, like a Kaplan-Meier plot, but specific to each patient, and “calibrated” – i.e., providing meaningful probability values. This motivates our PSSP system.

As shown in Figure A in S1 file, the PSSP system has two components: Shown vertically, the “PSSP-Learner” uses (historical) survival data (perhaps from a registry, where each row describes a patient, including the event time and censor bit) to select relevant variables, and then return a learned model over just those variables. The “PSSP-Predictor” then uses that model and a description of a novel patient, to produce a predicted survival distribution for that patient (this is shown, left to right).

Fig. A S1 file : **Basic Machine Learning approach.** Top-to-bottom: produce a PSSP model from a dataset of historical patients.  Left-to-right (across bottom): producing a survival curve for a novel patient, using a description of that patient (that includes only the selected variables), based on the learned PSSP Model.

**B. PSSP-Learning approach**

The PSSP-Learner first identifies a set of relevant variables; this process is discussed in the manuscript. It then, in essence, produces a set of logistic regression probabilistic predictors, one for each of a number of time points (here, we used r=53 $\approx$ $\sqrt{2769}$ time points). We sorted the subjects by the event time (whether it was a death, or last visit), then set the time points such that each interval included roughly the same number of subjects. This means the intervals are not evenly spaced.

The PSSP-Learner then combines these individual predictors in a way that ensures that the survival curve has to be monotonically decreasing. (We think of this as the “no zombie rule”: if someone dies, he stays dead.) See Yu et al. [1] for details.

**C. PSSP-Predictor**

The learned PSSP model is not simply a linear combination of the patient variables: it involves a weight for each patient variable at each of 53 timepoints { t_1_, ..., t_53_ }-- which means the learned model [
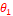
, ...,
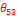
] itself involves 53 x (4+1) parameters (as well as 53 more, for the specific timepoints) to deal with the 4 patient features (and a bias term) -- ie, each
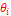
 has 4+1=5 parameters.  PSSP uses this model to produce a curve for each a patient, based on that patient’s 4 features, $f .$It first computes 53 values

  g( t_r_ ) = P( Dies $\geq t_{r} \left| f \right)= \frac{\left( \sum_{j=r}^{53} \exp\left( \sum_{i=j}^{53} f^{T} \theta_{i} \right) \right)}{\left( \sum_{j=1}^{53} \exp\left( \sum_{i=j}^{53} f^{T} \theta_{i} \right) \right)}$

-- one for each t_r_.  (Note the “r” on the first summation, corresponds to r-th timepoint).

This allows us to compute 54 (x, y) points { (0, 1), (t_1_, g(t_1_) ), (t_2_, g( t_2_) ), ... (t_53_, g( t_53_ ) ) }.

Our webtool then visualizes these points as a smooth curve (using splines).

That is, a variable might be very detrimental for the first year after LT, then become irrelevant for the next several years, and afterwards become protective. (This is why we did not remove variables that failed the assumption of time-independent hazards proportionality – ie, we did not run the Schoenfeld residuals test.)

This means that the curves for different patients can have very different shapes and can cross each other. Figure 2 in the main text and Figure B in S1 file each presents five representative curves, produced by applying our learned model to five patients. Technically, each curve is based on the model produced by a subset of 4/5 of the data, which did not include this patient, in a 5-fold cross-validation fashion [2].

Fig. B S1 file : **Example of individual survival curves**.

Each of the 5 solid lines corresponds to the survival distribution, produced by PSSP, of a single patient using the model including donor variables. The dashed curve is the Kaplan-Meier plot over the entire population (n=2769 patients).

**Evaluation Measures**

This Supporting Information section provides additional information about concordance, 1-calibration, and D-calibration, in general. The Supporting Information : « Evaluation of Measures » section provides statistics about how well PSSP performed, in terms of these measures.

**D. Concordance (discrimination measure)**

The concordance index (c-statistics) is commonly used in medicine to validate survival scores based on some risk model (often based on the Cox model). This concordance test, basically, considers each pair of “comparable” patients, and asks whether the predictor’s values for these patients, matches what happened to these two. In particular, if the model gives $x_{A}$ a “better” score than $x_{B}$, meaning it predicts that $x_{A}$ will live longer than $x_{B}$, then the model gets 1 point if this is what actually happens, for this ($x_{A}$, $x_{B}$) pair. If instead $x_{A}$ died before $x_{B}$, concordance would instead give this pair a 0. Concordance computes this for all pairs of comparable patients, and returns the average.

This measure is relevant when the goal is to rank patients -- e.g., when we want to know who will live longer between patients $x_{A}$and $x_{B}$. Hence, this concordance index can determine if a model can effectively prioritize patients according to urgency – i.e., deciding which patient will survive the least time, without a LT– see Step 2 of Figure 1 in the main text. Recall that our goal, here, is Step 1 (utility), which is different.

## E. Single-timepoint calibration (1-calibration)

Many research projects use 1-calibration to assess their survival model, which determines whether that probabilistic model is calibrated at a single specified time. We applied this measure to our learned PSSP model. First, we considered survival at 5 years. First, we considered survival at 5 years. We computed PSSP’s prediction $\hat{P}\left( 5\mathrm{years} \right|x_{i} )$ for each of the 2769 patients $x_{i}$. We then sorted these $\hat{P}\left( 5\mathrm{years} \right|x_{i} )$ values, and divided them in deciles — where the first decile includes the 277 ($\approx$ 2769 $\times$ 10%) patients with the largest $\hat{P}\left( 5\mathrm{years} \right|x_{i} )$ values, the second the next 10% with the “second” largest values, and so forth. For each such bin B, we then compute the average $ave(B)=\frac{1}{\left| B \right|}\sum_{x\in B} \hat{P}\left( 5\mathrm{years} \right| x)$. Here, this average is 0.726 for the first bin, which means we expect 0.726 $\times$ 277 $\approx$201 of these patients to be alive at 5 years. Simply removing censored patients from the calculation entirely biases the evaluation data. To account for patients censored before 5 years, we add “fractional” patients to our observations: if a patient $x_{A}$ is censored at $t_{A}$ < 5 years, we add an observation of KM(5 years)/KM($t_{A}$) to our number of patients surviving 5 years (where KM(t) is the Kaplan-Meier estimate of survival at time t). With these fractional observations included, we observed that 76.7% ($\approx$212) were alive. We did this for all 10 bins – see Figure 3B in the main text. We can evaluate this using the Hosmer-Lemeshow statistical test, whose null hypothesis is that the observed probabilities were random fluctuations around the expected values. Here, large p-values support the null hypothesis, suggesting the model is calibrated – i.e., should be used for this task.

**Distribution-Calibration**

**F. Motivation**

The text motivated the challenge of accurately estimating an individual patient’s survival probabilities, for several timepoints – e.g., for each of 3 months, 1 year, 5 years and 10 years. It then noted the standard measures are not designed to evaluate such a model: as this is a “calibration task” (providing estimates that have “semantic meaning” – here survival probabilities – of a single patient), rather than a “discriminative measure” (which is based on the *relative* survival times of pairs of patients), the standard discriminate measures, such as concordance index, are not relevant. We also noted that the standard *calibration* measure, called “1-calibration” in the text, also does not match our criteria, as it is designed to deal with only *a single time point*, but our goal is for multiple time points.

Given our goal, we realized the appropriate model is to produce a complete “survival distribution” for each patient. This means we need an evaluation measure that can determine if the entire *set of distributions* is calibrated, leading to our definition of “Distribution-calibration” (D-calibration). Below we explain the measure, starting with a simplified model.

As a thought experiment, imagine 100 individuals appeared identical (i.e., had the same values for the recorded variables), and each received an apparently-identical LT at the same time. Of course, we cannot expect these patients to die at the same moment, as we anticipate their post-operative courses will be stochastic, influenced by many factors that are not included in the database (e.g., post-operative complications, infections, different immunosuppression, rejection episodes, etc.). This mean we cannot predict the exact date of death, but we can predict a *survival distribution*, giving the survival probability $\hat{P}(t)$ for every post-transplant time – say the red curve shown in Fig. C1 in S1 file, which plots $\hat{P}\left( t \right)$ versus t.

Now imagine the first patient dies at 6 months post-transplant (green vertical line in left part of Fig. C1 in S1 file). Note $\hat{P}(6\mathrm{months})$= 0.83. As this value is in the interval [0.75, 1.00], this patient dies in the first predicted quartile of the survival distribution. A rectangle corresponding to the percentage of the patient (in the present example 1/100 = 1%) is added in the quartile [75,100] of a sideways histogram (right part of Fig. C1 in S1 file). If the second patient dies at 3.2 years post-LT, as $\hat{P}( 3.2\mathrm{years})$ = 0.59, a second square is added in its corresponding quartile of the same histogram (Fig. C2 in S1 file). Now imagine the 3^rd^ and the 4^th^ patients die respectively at 4.4 years and 6.3 years post-transplant; this would add two other 1%-squares to the appropriate quartiles of the histogram (Fig. C3 in S1 file). If the 5^th^ patient dies at 3.5 years post-LT, a further 1%-square would be added in the [50,75] quartile, and so forth (Fig. C4 in S1 file).

If the model is perfectly calibrated, we expect that 25% of the patients die in each quartile – which here means 25 of the 100 patients considered (Fig. C5 in S1 file).

Fig. C1 to C6 S1 file: **Step-by-step example to illustrate the concept of distribution-calibration**

**G. Censored patients**

The above method only deals with uncensored patients – i.e., those whose time-of-death is known. However, most of the PSC patients are alive at the time of the last follow-up. For those censored patients, the quartile of the death event is unknown – instead, we only know that they were alive in the quartile corresponding to the probability of the time of that last meeting. To incorporate this information, we make the standard assumption that the time of the event (death) is independent of the time of measurement [3].

Consider first a patient who was censored at 3.5 years post-LT (left of Figure C6 in S1 file), which the learned model predicts as 55.5% probability. As this patient is still alive in the quartile [50,75], this independence assumption means that he has 10% risk of dying in the current quartile [50,75] (as 5.5% of the 55.5% is in this quartile, and 5.5/55.5 ≈ 0.10), a 45% risk of dying in the quartile [25,50] (as 25% of the 55.5% is in this quartile, and 25/55.5 ≈ 0.45) and a 45% risk of dying in the quartile [0,25]. This means adding a 0.1% block to the [50,75] quartile, and 0.45% blocks to each of the [25,50] and [0,25] quartiles; see right of Figure C6 in S1 file.

Now consider a different patient, who was censored at the end of the first quartile (say after 1.9 year); this would require adding 0.33 to each of the quartiles [50,75], [25,75] and [0,25]; and so forth. Again, if the model if perfectly D-calibrated, the total in every square (from both censored and uncensored patients) should correspond to 25%.

However, even a perfect *quartile* D-calibration might not be enough to accurately measure the precision of a tool for predicting post-transplant survival. Rather than divide the survival probability in 4 quartiles, we instead use 10 bins, and ask whether each corresponds to 10% of the population. This leads to the histograms shown in main text Fig. 3A and Fig. D in S1 file.

Fig. D S1 file: **Sideways histogram, to visualize D-calibration of the model including donor variables.**

The “p-value” here (0.999) is the result of the $\chi^{2}$test, on these values.

In practice, of course, patients will not be identical, but will have different values for the observed variables leading to 100 different predicted survival curves (perhaps 5 of which appear in Figure B in S1 file). We can still use the same method to produce a histogram, to evaluate the D-calibration of this heterogeneous population. In our situation, we actually have 2769 patients, rather than 100, which means each 1/100 above would instead be 1/2769.

## H. Validating if a model is D-Calibrated

We consider a model to be useful for our screening test if it is D-calibrated. To determine whether a given PSSP model is D-calibrated, we used a χ^2^-test to evaluate the chance that the observed distribution (D_o_, here represented graphically by a sideways histogram) was generated by a uniform distribution, which is the null hypothesis. Here, we are considering 10 bins, which means we expect each bin to contain 10% of the population. A model is good if we fail to reject that null hypothesis, which means large P-values suggest the model is good.

To conclude this comparison, we note that there are many similarities between 1-calibration and D-calibration, as each deals with a model that assigns probabilities to each patient, and considers bins corresponding to deciles of the population. They differ in that 1-calibration …

- considers a single time $\hat{P}\left( 5\mathrm{years} \right|x_{i} )$, while D-calibration deals with the entire distribution over time $\hat{P}\left( t \right|x_{i} )$ for all $t\geq0$;
- matches the empirical percentage of death (in each decile), while D-calibration matches the uniform distribution;
- uses the Hosmer-Lemeshow test, while D-calibration uses the $\chi^{2}-test.$

## I. Further Comments about Evaluation Measures

We discussed several measures for evaluating a survival analysis/prediction system: concordance, 1-calibration and D-calibration. For each, we should of course evaluate the quality of a model by computing the score on a “test set”, which is disjoint from the data on which this model was initially learned. All of the results reported here are based on a version called “5-fold cross-validation”: This involves running all of the steps (both variable selection, then training) on 4/5 of the training instances to produce a model, then evaluating the resulting model by running it on the held-out 1/5 of the data [2]. We then report the average statistics, over these 5 runs.

Also, there are issues that arise due to the censored patients: We can only run the concordance index test on the “comparable” pairs of subjects, where we can determine which one died first – e.g. if both are uncensored, or if a censored subject was censored after an uncensored patient died.

**J. Another Related PSSP Model**

Due to space constraints, the main text focused on a predictor having access to information about transplant candidates only (which is the situation at the time of wait-listing). We will refer to this as the Tx1-D model. This Supporting Information section considers another model, Tx1+D, which also includes information about the donor (which is the situation at the time of organ offer).

Including donor variables can improve the models in two situations: 1. at the time of organ offer, to assess the utility of a combination recipient/donor, and 2. at the time of wait-listing, to simulate the best expected outcome in case of good donor or a worse outcome in case of poor quality organ. We used the same PSSP-learning process, but here ran this on a dataset that extended the recipient data with information about the donor. Our learning algorithm consider all of the recipient variables shown earlier, and also the following donor/graft variables, all at time of LT: donor age, donor gender, donor weight, donor height, donor ABO Group, ABO compatibility, donor type (cadaveric/living), donor type (brain dead/cardiac dead), donor cause of death, and the centre’s experience in living donor transplants (>15 versus < 15 such operations) .

Due to the different policies in organ acceptance between different centres, we can expect different types of donors even for similar recipients. This variability is important for PSSP, as an effective learned model needs access to the appropriate range of training instances, which here means observing the outcome in similar patients with different profiles of donors.

For the Tx1+D model, the variable selection process happened to select the same variables it found for the recipient-only model (Tx1-D), and also two other donor-related variables: donor type (cadaveric/living) and donor height.

The donor type (cadaveric versus living) has already been studied in transplantation for PSC, showing a better post-transplantation survival with living donors compared to cadaveric donors [4]. A higher donor height has also been associated with better post-LT survival [5].

As the centre experience in living donors liver transplantations (LDLT) has been associated with post-transplantation survival [6], we further analysed if the center experience biaised the results. We therefore first identified all the centers that performed more than 15 LDLT between 1998 and 2002 in the whole SRTR database, for any transplant indication - PSC or other - using the inclusion and exclusion criteria presented in the Methods section (15 centers, 872 patients). We compared, in a univariate model, the effect of the center experience in LDLT on post-transplantation survival. We found there was no difference in survival after LDLT between centers with versus without the experience of more than 15 cases (p=0.872).

Our on-line calculator <http://pssp.srv.ualberta.ca/calculator/liver_transplant_2002> allows the user to make predictions based on either models – either Tx1-D (recipient variables only) and Tx1+D (both recipient and donor variables).

**Evaluation of Measures**

The main text provided evaluations for the Tx1-D model, in terms of 1-calibration and D-calibration. Here, we also show its quality in terms of concordance. We also evaluate the Tx1+D model, which also includes the donor variables. These results are summarized in the Table 1 in S1 file.

Table 1 S1: **Concordance and discrimination of the different models**.

|  | PSSP model with donor information | Cox-KP model with donor information | PSSP model without donor | Cox-KP model without donor information |
| --- | --- | --- | --- | --- |
| D-Calibration | 1.000 | 1.000 | 1.000 | 1.000 |
| 1-calibration 3mon | 0.150 | 0.296 | 0.291 | 0.375 |
| 1-calibration 1yr | 0.527 | 0.353 | 0.129 | 0.322 |
| 1-calibration 5yr | 0.632 | 0.150 | 0.278 | 0.189 |
| 1-calibration 10yr | 0.678 | 0.026 | 0.409 | **0.027** |
| Concordance | 0.590 | 0.596 | 0.582 | 0.588 |

Summary of the discrimination (Concordance) and calibration (1-calibration, D-calibration) tests for PSSP and Cox-Kalbfleisch-Prentice models, with and without donor information. Recall a higher p-value means the model is better.

**K. Concordance**

Standard risk models provide a single number for each patient, which can be used for the comparisons needed to compute the concordance score. As PSSP returns an entire curve for each subject, we need to decide how to obtain a single number. Here, we decided to the use the patient’s mean survival time (which is the area under the survival curve). (We actually use the negative of this, as we want larger values to suggest earlier time of death.) The concordance index here was 0.582 for Tx1-D and 0.590 for Tx1+D; see Table 1 in S1 file. While these are better than chance, we cannot recommend prioritizing organ allocation to maximize urgency based on our model solely. This is not problematic, as this measurement is not relevant for our “utility” goal here.

**L. 1-Calibration**

For the model including donor variables, the 1-calibration (Hosmer-Lemeshow) p-values at 3 months, 1, 5 and 10 years, were: 0.150, 0.527, 0.632 and 0.678 respectively.

**M. D-calibration**

Figure D in S1 file presents the D-calibration histograms for the model with donor information.

**References**

1. Yu C-N, Greiner R, Lin H-C, Baracos V. Learning patient-specific cancer survival distributions as a sequence of dependent regressors. In: J. S-T, R.S. Z, Bartlett PL, Pereira F, Weinberger KQ, eds. *Advances in Neural Information Processing Systems*, 2011: 1-9.

2. Witten IH, Frank E, Hall M. *Data Mining: Practical Machine Learning Tools and Techniques*. 3rd edn. Morgan Kaufmann, 2011; 664.

3. Klein J, Moeschberger M. *Survival Analysis: Techniques for Censored and Truncated Data*. Springer, 2005.

4. Kashyap R, Safadjou S, Chen R, et al. Living donor and deceased donor liver transplantation for autoimmune and cholestatic liver diseases--an analysis of the UNOS database. *J Gastrointest Surg*. 2010; **14**: 1362-9.

5. Feng S, Goodrich NP, Bragg-Gresham JL, et al. Characteristics associated with liver graft failure: the concept of a donor risk index. *American journal of transplantation : official journal of the American Society of Transplantation and the American Society of Transplant Surgeons*. 2006; **6**: 783-90.

6. Olthoff KM, Abecassis MM, Emond JC, et al. Outcomes of adult living donor liver transplantation: comparison of the Adult-to-adult Living Donor Liver Transplantation Cohort Study and the national experience. *Liver Transpl*. 2011; **17**: 789-97.

Fig. A S1

Fig. B S1


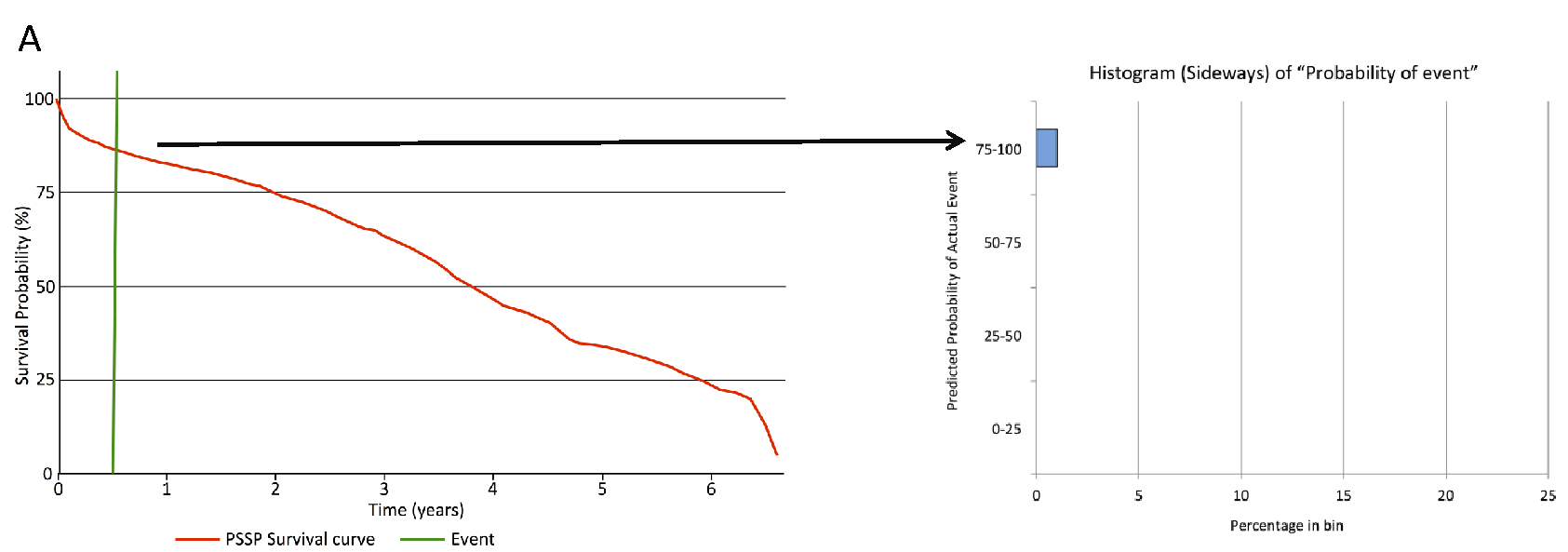


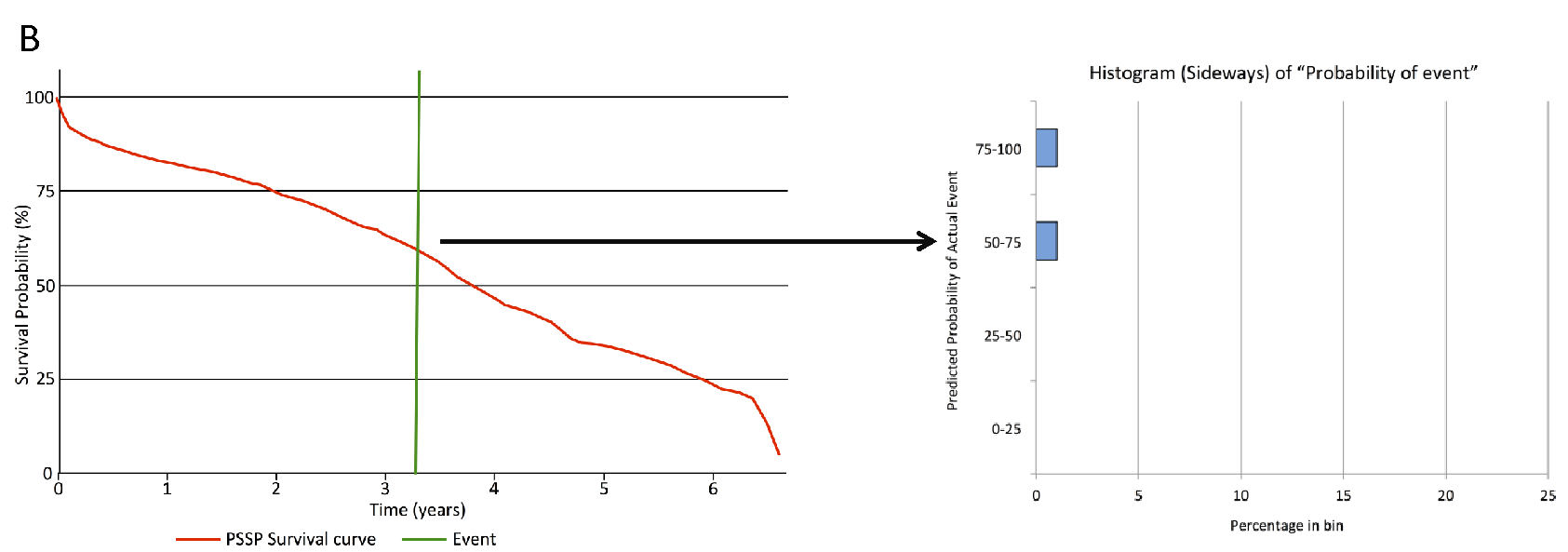


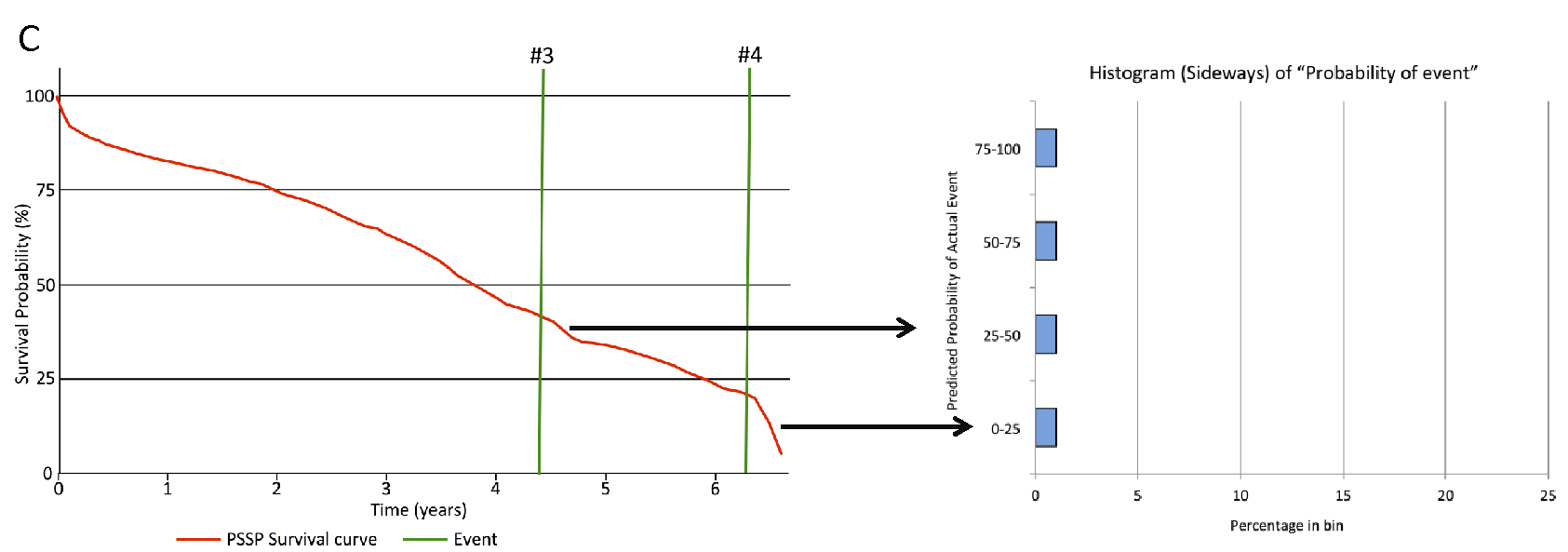


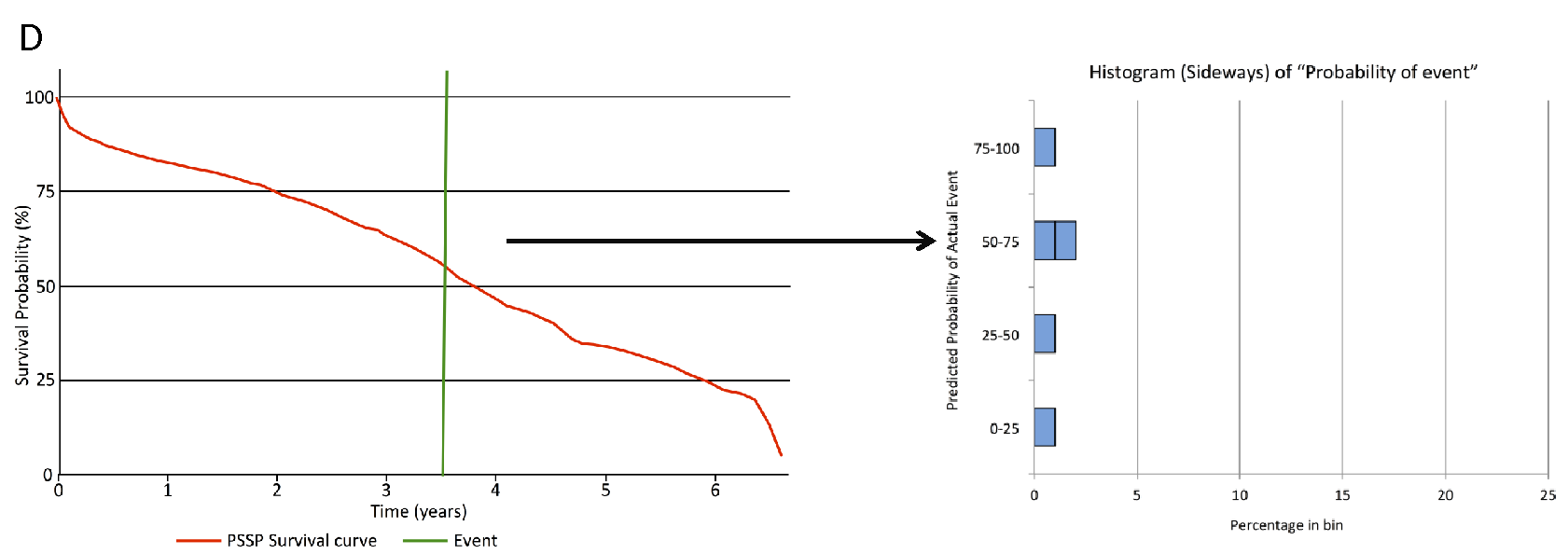


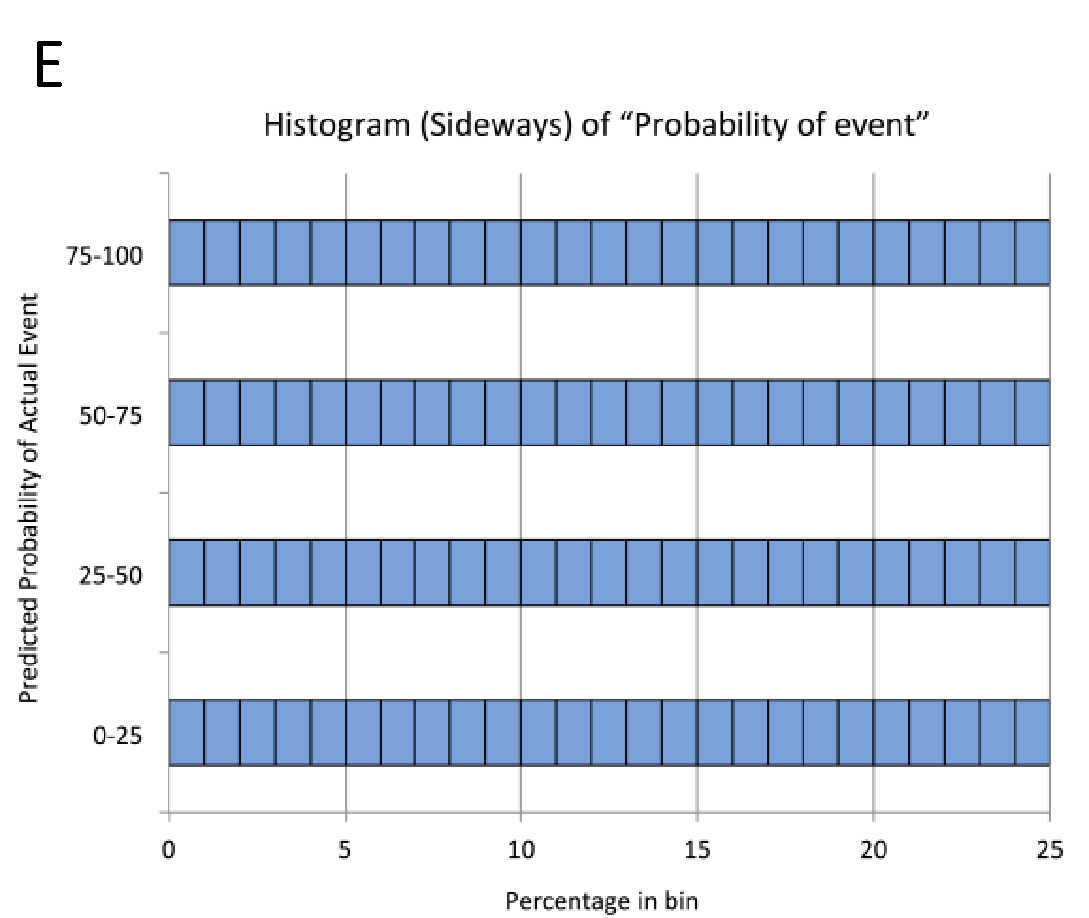


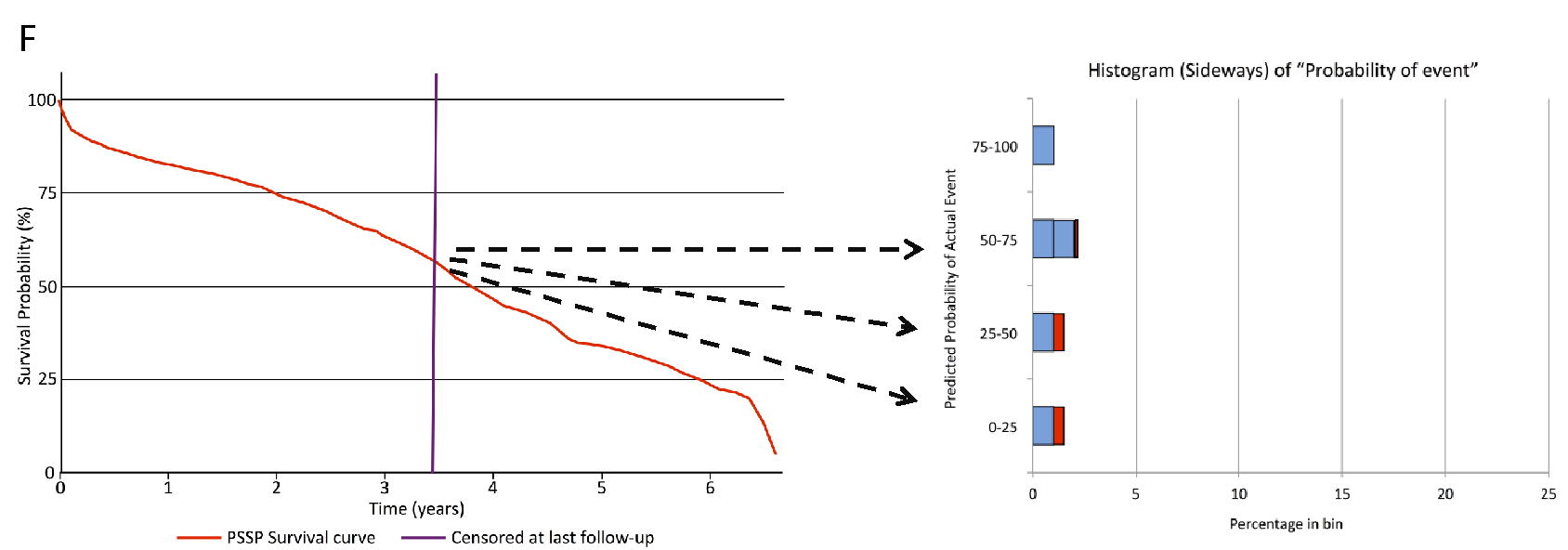


Fig. C1 to C6 S1

Fig. D S1
